# Set binding factor 1 (SBF1/MTMR5) as a potential novel target in colorectal cancer: an exploratory immunohistochemical and bioinformatics study

**DOI:** 10.1590/1414-431X2026e15598

**Published:** 2026-10-12

**Authors:** A. Alatawi, I. Alhabib, M. Fagere, F.A. Al-Jindan, K. Alsamman, F.M. Alzahrani, A.Y. Owaidah, O.S. El-Masry

**Affiliations:** 1 Department of Clinical Laboratory Sciences, College of Applied Medical Sciences, Imam Abdulrahman Bin Faisal University, Dammam, Saudi Arabia; 2 Department of Pathology and Laboratory Medicine, College of Medicine, Imam Abdulrahman Bin Faisal University, Dammam, Saudi Arabia; 3 Princess Aljawhara Al-Ibrahim Center for Molecular Medicine, Genetics and Inherited Disorders, Molecular Medicine Department, College of Medicine and Health Sciences, Arabian Gulf University, Manama, Kingdom of Bahrain

**Keywords:** Colorectal cancer, SBF1, MTMR5, Phosphoinositide, Bioinformatics, Biomarkers

## Abstract

Colorectal cancer (CRC) is a major cause of cancer-related morbidity and mortality worldwide, and dysregulation of phosphoinositides signaling pathways plays a critical role in its pathogenesis. The involvement of Set Binding Factor 1 (SBF1; MTMR5), a pseudophosphatase of the myotubularin family in CRC, remains unexplored. This study compiled experimental and bioinformatics approaches to investigate the expression and potential relevance of *SBF1* in CRC. Immunohistochemical analysis was performed on CRC tissue specimens and corresponding normal adjacent tissues (NAT), with estimation of SBF1 staining followed by statistical comparison. In parallel, bioinformatics assessed gene ontology and protein-protein interactions, and open CRC datasets were analyzed to assess *SBF1* alterations, expression profiles, and survival associations. The results demonstrated a significant overexpression of *SBF1* in CRC tissues compared with NAT. Bioinformatics revealed SBF1 as a partner within a functional network enriched in phosphoinositide metabolism. Bioinformatics identified *SBF1* genetic alterations in approximately 5% of cases, and survival analysis indicated that wild-type *SBF1* could be an unfavorable prognostic marker, as reflected by hazard assessment. Collectively, these findings provided the first experimental evidence that *SBF1* is overexpressed in CRC and suggest that it may represent an unrecognized component of phosphoinositide related pathways involved in colorectal tumorigenesis.

## Introduction

Colorectal cancer (CRC) is one of the most frequently diagnosed malignancies worldwide and represents a major cause of cancer-related mortality. According to global cancer statistics, CRC ranks as the third most commonly diagnosed cancer and the second leading cause of cancer-associated death (1). In Saudi Arabia, CRC is the second most prevalent cancer, accounting for a substantial proportion of newly diagnosed malignancies and demonstrating a rising incidence in recent years (2). Epidemiological factors such as age, sex, and geographical location strongly influence CRC incidence and outcomes, reflecting the complex interplay between genetic susceptibility and environmental exposures (3).

The etiology of CRC is multifactorial and involves both environmental and genetic risk factors. Environmental contributors include obesity, physical inactivity, smoking, alcohol consumption, and dietary patterns characterized by low fiber intake and high red meat consumption, all of which have been associated with increased CRC risk (4,5). Genetic predisposition also plays a significant role, particularly in hereditary cancer syndromes such as familial adenomatous polyposis and Lynch syndrome, also known as hereditary nonpolyposis CRC (3). These factors collectively contribute to colorectal tumorigenesis through cumulative molecular alterations that disrupt normal cellular homeostasis.

At the molecular level, CRC develops through a stepwise accumulation of genetic and epigenetic changes that affect key regulatory pathways controlling cell proliferation, differentiation, and survival. Three major molecular mechanisms have been identified as primary drivers of CRC initiation and progression: chromosomal instability (CIN), microsatellite instability (MSI), and the CpG island methylator phenotype (CIMP) (6). CIN, the most prevalent molecular risk, accounts for approximately 80-85% of CRC cases and is characterized by large scale chromosomal aberrations, loss of heterozygosity, and structural rearrangements that alter the function of tumor suppressor genes and oncogenes, including *APC*, *KRAS*, and *TP53* (7). MSI results from defects in DNA mismatch repair mechanisms, leading to accumulation of mutations within microsatellite regions and subsequent disruption of genes involved in growth regulation (8). In contrast, CIMP is driven by widespread promoter hypermethylation of CpG islands, resulting in transcriptional silencing of tumor suppressor genes and promotion of malignant progression (8).

Despite the clinical utility of traditional staging systems such as TNM classification, these approaches do not fully capture the biological and molecular heterogeneity of CRC. To address this limitation, consensus molecular subtypes (CMS) have been developed based on gene expression profiling, categorizing CRC into four biologically distinct subtypes with differing prognostic and therapeutic implications (9). These classifications underscore the importance of understanding intracellular signaling networks that contribute to tumor behavior beyond conventional histopathological criteria.

Among the signaling pathways implicated in CRC, phosphoinositide-dependent signaling plays a central role in regulating cell growth, survival, and metabolism. Phosphoinositides are minor but functionally critical membrane lipids that act as signaling transducers through dynamic phosphorylation and dephosphorylation of the inositol ring (10). Dysregulation of phosphoinositide metabolism has been strongly linked to CRC pathogenesis, particularly through aberrant activation of the PI3K-PI(3,4,5)P_3_-Akt pathway (11). Hyperactivation of this pathway promotes uncontrolled proliferation, resistance to apoptosis, and tumor progression, while its negative regulation by lipid phosphatases is essential for maintaining cellular homeostasis (12). In this context, phosphatase and tensin homolog (PTEN) is a key tumor suppressor that counteracts PI3K activity by dephosphorylating PI(3,4,5)P_3_, thereby limiting Akt activation (12). Loss or inactivation of PTEN has been reported in multiple cancer types, including CRC, highlighting the importance of lipid phosphatases in tumor suppression (13). In addition to PTEN, other phosphoinositide-regulating enzymes, such as myotubularins (MTMs), have emerged as potential modulators of oncogenic signaling, although their roles remain less well characterized.

MTMs represent a conserved family of phosphoinositide phosphatases that selectively dephosphorylate PI3P and PI(3,5)P_2_, regulating endosomal trafficking, membrane dynamics, and receptor signaling (14). Beyond their established functions in membrane trafficking, MTMs have been implicated in cellular processes relevant to cancer, including proliferation, survival, and apoptosis (15). Notably, alterations in the expression or activity of specific MTM family members have been reported in CRC, suggesting a context-dependent role in tumor progression (16).

Set Binding Factor 1 (SBF1), also known as MTMR5, is a catalytically inactive member of the MTM family classified as a pseudophosphatase due to mutations within its phosphatase domain (17). Despite lacking enzymatic activity, SBF1 retains multiple conserved domains and functions as a regulatory adaptor that interacts with catalytically active MTMs, particularly MTMR2, enhancing their activity and influencing subcellular localization (18). SBF1 has also been proposed to regulate membrane trafficking through Rab GTPase activation, implicating it in intracellular transport processes that are increasingly recognized as relevant to cancer biology (19).

We have recently reported the downregulation of *SFB1* in chronic myeloid leukemia (20). To date, the association between SBF1 and CRC remains poorly defined, with limited experimental evidence addressing its expression and functional significance in colorectal tumors. Given the critical role of phosphoinositide signaling in CRC pathogenesis and the emerging regulatory functions of MTMs, investigating *SBF1* expression in CRC tissues represents a scientifically justified objective. The present study, therefore, aimed to evaluate the expression profile of *SBF1* in colorectal tumor tissues compared with normal adjacent tissues, providing experimental insights into its potential involvement in colorectal tumorigenesis. Additionally, bioinformatics analysis was performed to understand the molecular function of SBF1, the biological processes in which it is involved, and its potential prognostic value in publicly available cancer genomics datasets.

## Material and Methods

### Gene ontology and protein-protein interaction analysis

All bioinformatics analyses were performed using publicly accessible web-based platforms. The Gene Ontology (GO) annotations were retrieved from the Gene Ontology Consortium database (http://geneontology.org/) using the human SBF1 gene identifier (Gene ID: 6305). GO terms corresponding to biological process, molecular function, and cellular component were collected using the default annotation filters provided by the database (21,22). Protein-protein interaction analysis was conducted using the STRING platform (https://string-db.org/) with the organism restricted to *Homo sapiens*. Interaction networks were generated using the STRING default parameters with a minimum interaction score of 0.4 (medium confidence) and including evidence from curated databases, experimental data, and co-expression sources (23). Pathway enrichment analysis was further explored using the Reactome database (https://reactome.org/) by querying the *SBF1* gene within the human pathway browser to identify reactions and molecular pathways involving SBF1 (24). All databases were accessed on June 22, 2025, and analyses were performed using the built-in analytical tools provided by each platform without modification of the raw genomic datasets.

### Survival analysis and tissue expression using the Human Protein Atlas

Survival and gene expression analyses were conducted using the Human Protein Atlas (HPA) platform (https://www.proteinatlas.org/) (25). *SBF1* expression profiles across normal tissues and cancer types were explored using the HPA RNA-seq datasets derived from The Cancer Genome Atlas (TCGA) and other publicly available transcriptomic resources integrated within the HPA portal. Kaplan-Meier survival plots were generated using the built-in survival analysis module of the HPA database, where patients were automatically stratified into high- and low-expression groups based on the median expression level of *SBF1* as defined by the platform. Overall survival was evaluated using the log-rank test implemented within the HPA analytical framework. In addition to transcriptomic data, immunohistochemical protein expression images available within the HPA tissue atlas were examined to evaluate SBF1 protein localization and expression patterns in normal and malignant tissues. All analyses were conducted using the default parameters provided by the HPA portal, and the database was accessed on June 22, 2025.

### Gene expression profile and variant analysis in public CRC datasets

Genomic alteration analysis was performed using the cBioPortal platform (https://www.cbioportal.org/) (26), an interactive resource that integrates multidimensional cancer genomics datasets from large-scale sequencing projects. The query was restricted to the human *SBF1* (*MTMR5*) gene across CRC studies available within the portal at the time of analysis. A combined dataset consisting of 17 CRC studies comprising approximately 6,235 tumor samples from 6,109 patients were analyzed using the cBioPortal “Query by Gene” function. The analysis evaluated somatic mutations, copy-number alterations, and structural variants affecting the *SBF1* gene. Alteration frequencies and mutation types were extracted using the OncoPrint visualization tool. Survival analyses comparing cases with *SBF1* alterations versus unaltered (wild-type) cases were performed using the built-in Kaplan-Meier survival module of cBioPortal, which calculates overall survival using the log-rank test and estimates hazard ratios using a Cox proportional hazards model. All analyses were conducted using the default parameters provided by the cBioPortal interface, and the database was accessed on June 22, 2025. The specific links to CRC studies included in the combined analysis are deposited in Figshare repository and can be accessed at https://doi.org/10.6084/m9.figshare.31491037 along with the combined dataset link.

### Patient samples and tissue specimens

This laboratory-based, cross-sectional study investigated the expression and localization of SBF1 in CRC tissues compared with normal adjacent tissues (NAT) and was approved by the Institutional Review Board at Imam Abdulrahman bin Faisal University and the Department of Internal Medicine at King Fahd University Hospital (IRB-2024-01-583).

### Tissue collection and clinical data

A total of fifty formalin-fixed, paraffin-embedded CRC tissue sections and their corresponding normal adjacent tissues were collected from King Fahd University Hospital at Imam Abdulrahman bin Faisal University between June and August 2025. Sample size was determined based on feasibility estimation by the appointed clinical pathologist. Available clinicopathological data included patient age, sex, tumor anatomical site, diagnosis, and tumor stage; these parameters were documented and analyzed in the Results section.

### Immunohistochemical detection of SBF1

Paraffin-embedded sections were deparaffinized by immersion in two consecutive changes of xylene for 2 min each. Rehydration was performed through a graded ethanol series (100, 90, and 70%) followed by rinsing in distilled water to complete hydration. Heat-induced antigen retrieval was performed using trisodium citrate buffer (pH 6.0). Slides were immersed in freshly prepared buffer, heated using a microwave protocol, and the remaining protocol steps were followed as previously described (27). Sections were then incubated overnight at room temperature in a humidified chamber with mouse monoclonal anti-MTMR5 (SBF1) antibody (1:100 dilution) (Santa Cruz, Cat# sc-393488, USA). The following day, slides were washed with TBST and incubated with horseradish peroxidase (HRP)-conjugated goat anti-mouse secondary antibody (1:1000 dilution) (Molequle-On; Cat# AB-M-009, New Zealand) for 1 h at room temperature in a humidified chamber.

Signal visualization was achieved using a 3,3'-diaminobenzidine (DAB) substrate kit (Cat# DAB-M-3; Molequle-On). Sections were incubated with DAB substrate in the dark to generate a brown reaction product, followed by counterstaining with hematoxylin. Slides were dehydrated through ascending ethanol concentrations, cleared in xylene, and mounted using an aqueous mounting medium and coverslips were then applied.

### Immunohistochemical scoring

Stained slides were independently evaluated by two pathologists. Signal intensity and staining patterns were assessed to determine SBF1 expression levels in CRC tissues compared to normal adjacent tissues. Patient characteristics and raw data of stain intensity scoring are publicly available through https://doi.org/10.6084/m9.figshare.31500085.

### Statistical analysis

Quantitative data are reported as means±SE. Comparisons between CRC tissues and normal adjacent tissues were performed using paired *t*-test. Pearson's correlation was used to assess the correlation between the numerical tumor stage and SBF1 staining scores. Moreover, the association between CRC stage class and staining score class was assessed by the Fisher's exact test. Statistical significance for survival analysis and hazard ratio were calculated using the built-in software of bioinformatics portals. Statistical analyses were performed using GraphPad Prism software (version 10.6.1; USA), and P values ≤0.05 were considered statistically significant.

## Results

### Gene ontology, protein-protein interaction (PPI) network, and pathway enrichment of *SBF1*

STRING database showed that *SBF1* interacts with ten proteins, forming a biologically connected interaction network (Figure 1). The PPI enrichment analysis yielded a statistically significant enrichment P-value of 0.00561, indicating that these proteins are functionally linked rather than randomly associated. GO enrichment analysis revealed that the interacting protein network is primarily associated with phosphatidylinositol dephosphorylation, phosphatidylinositol biosynthetic processes, and regulation of phosphoinositide metabolism. The dominant molecular functions included phosphatase activity, particularly phosphatidylinositol-3-phosphate and phosphatidylinositol-3,5-bisphosphate phosphatase activities. These processes collectively implicate SBF1 in phosphoinositide-regulated signaling pathways (Supplementary Table S1).

**Figure 1:**
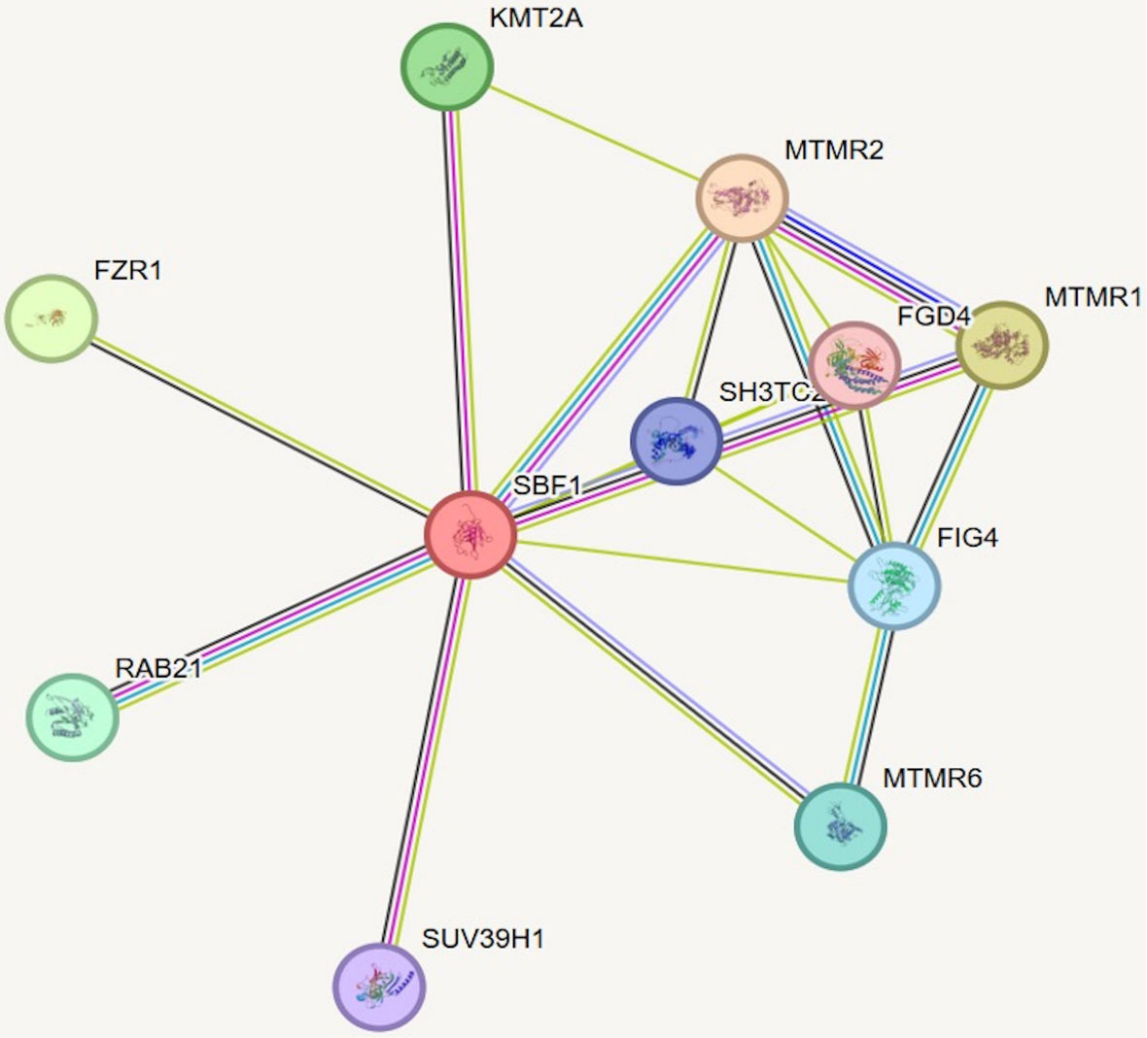
Protein-protein interaction (PPI) network of SBF1. SBF1 (MTMR5) interacts with 10 other proteins to achieve its molecular functions in different biological processes including its interaction with other MTMRs (MTMR1, 2, and 6). The colored lines connecting proteins represent different types of evidence supporting the PPI, including experimentally validated interactions, curated database associations, co-expression data, and predicted functional links, as defined by the String database. The figure was provided by String Portal (23).

Detailed analysis of SBF1 interaction partners demonstrated strong associations with several proteins involved in intracellular trafficking, signaling, and epigenetic regulation. High-confidence interactions (score≥0.7) were observed with MTMR2, MTMR1, FZR1, KMT2A, and RAB21. These interactions suggest that SBF1 participated in phosphoinositide signaling, vesicle trafficking, cell cycle regulation, and epigenetic modulation through its functional network (Supplementary Tables S2 and S3). This analysis demonstrated a direct association of *SBF1* network with regulatory functions related to cell proliferation and survival and regulation of gene expression; these are crucial molecular processes in tumorigenesis.

Reactome pathway analysis showed that SBF1 interacts with MTMR2 forming SBF1-MTMR2 complex that dephosphorylates PI(3,5)P2 to PI5P and PI3P to PI. Additional analysis showed that SBF1 interacts with 7 other proteins (Figure 2).

**Figure 2:**
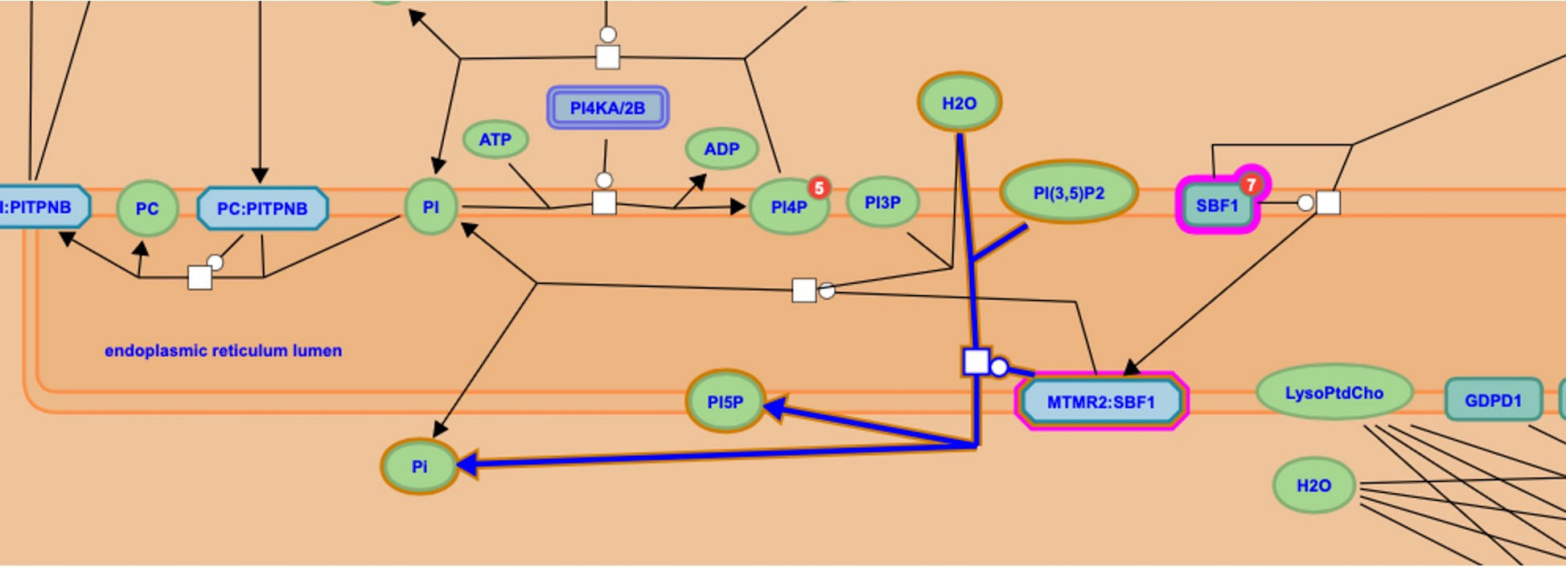
Reactome pathway illustration for SBF1. SBF1 functions through the formation of a complex with MTMR2, which in turn dephosphorylates PI(3,5)P2 to PI5P and PI3P to PI. SBF1 has 7 other interactions, indicating its involvement in several cellular pathways. The figure was adapted from the Reactome portal (24).

### SBF1 in the Human Protein Atlas

Survival analysis of TCGA datasets provided by the Human Protein Atlas (25) indicated that SBF1 is a potential favorable prognostic marker in kidney renal papillary cell carcinoma (RPCC), where high expression was significantly associated with better survival probabilities (P=0.00018) (Figure 3A). On the other hand, high *SBF1* expression was associated with worse overall survival probabilities in liver hepatocellular carcinoma (LIHC) patients being a significant unfavorable prognostic marker in this cancer (P=0.00046) (Figure 3B). It should be noted that survival analysis from the Human Protein Atlas did not report a significant prognostic utility of SBF1 in CRC, although there was a significant difference in survival probabilities between low and high expression groups in colon adenocarcinoma (P=0.021). This lack of clinical relevance might be due to the heterogeneity of the analyzed cohort or the strict threshold for significance set by the portal. Therefore, other cancer types were explored to assess the broader prognostic relevance of SBF1, where its expression was significantly associated with survival analysis.

**Figure 3:**
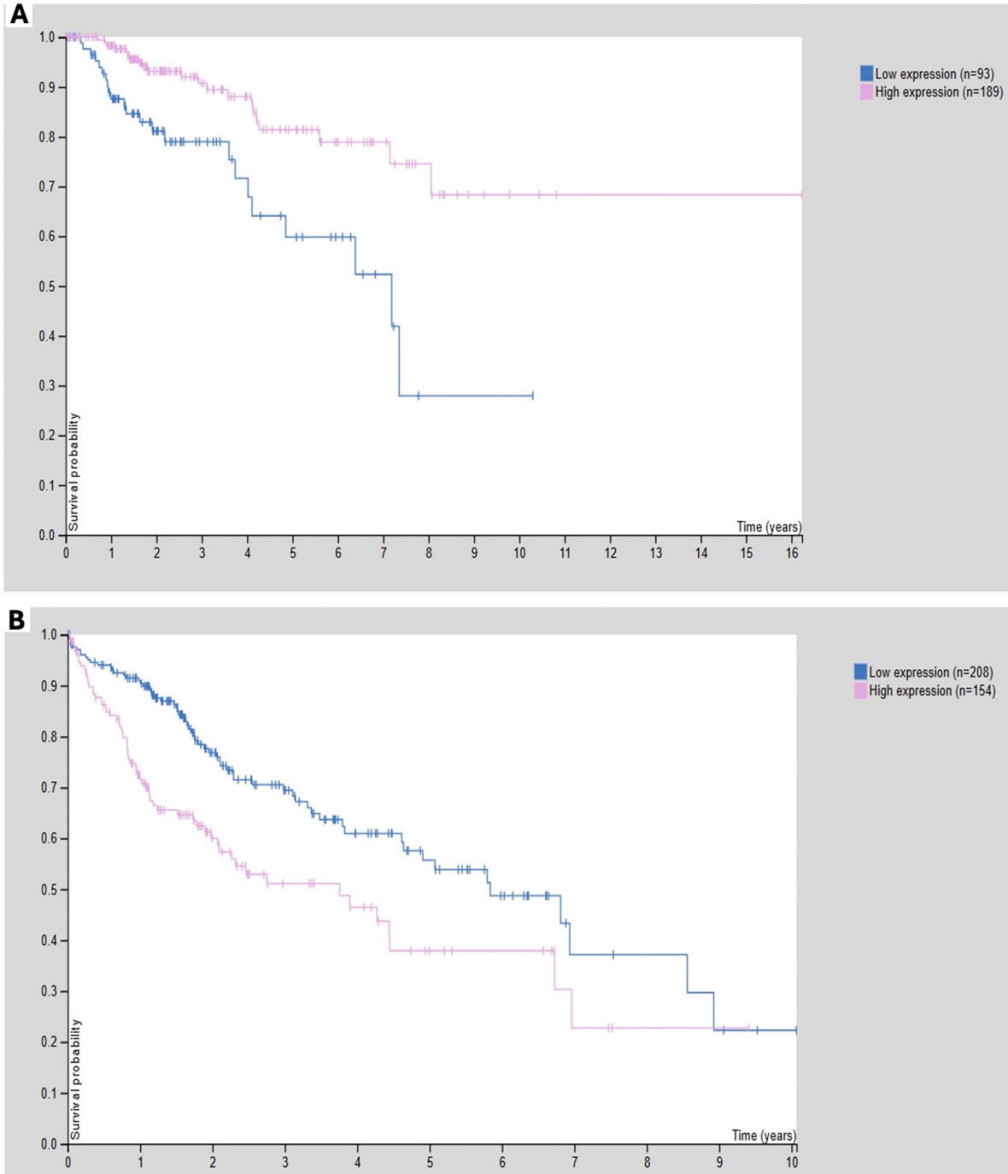
Kaplan-Meier curves showing the survival analysis of renal papillary cell carcinoma (PRCC) and liver hepatocellular carcinoma (LIHC) patients from the Human Protein Atlas based on *SBF1* expression levels. *SBF1* was a favorable marker in PRCC (P=0.00018) and an unfavorable marker in LIHC (P=0.00046). Kaplan-Meier plots were created with the Human Protein Atlas built-in tools (25).

### Genetic alterations and expression profile of *SBF1* in cBioPortal CRC datasets

Publicly available CRC datasets were analyzed using the cBioPortal platform (26) to characterize *SBF1* genetic alterations. Analysis of 7,092 CRC samples across 17 studies revealed that *SBF1* was genetically altered in approximately 5% of cases (Supplementary Figure S1).

Among these alterations, somatic mutations constituted the majority (68%), while deep deletions accounted for 32% of detected events. A total of 192 somatic mutations were identified in CRC datasets, including 105 missense mutations, 23 truncating mutations, and 64 structural variants. Several recurrent frameshift and splice-site mutations were reported, including T810M, X881 splice, S916Qfs*11, S1077Qfs*11, S1077Afs*28, and G915Afs*47 variants. Since detailed annotations of these mutations are not provided by the cBioPortal platform, nor the NCBI dbVar portal, the biological and oncogenic significance of these mutations remains undetermined. They are reported descriptively to highlight their presence in CRC populations. The relationship between *SBF1* genetic alterations and protein expression levels was not directly assessed experimentally in this study and remains to be investigated.

### Survival analysis of *SBF1* alterations in CRC

Kaplan-Meier survival analysis was conducted using cBioPortal data to evaluate the association between *SBF1* genetic alterations and overall survival in CRC patients. Patients were divided into two groups: altered *SBF1* group (n=106) and unaltered (wild-type) *SBF1* group (n=2,344). At 60 months, overall survival probability was approximately 78% in the altered group compared to 60% in the unaltered group, indicating an apparent survival advantage for patients harboring *SBF1* alterations. The log-rank test produced a P-value of 0.0791, indicating that this relationship did not reach statistical significance. However, hazard ratio analysis showed that patients with unaltered *SBF1* had a significantly higher risk of death compared to those with altered *SBF1* (HR=1.460; 95%CI: 1.019-2.093), suggesting that *SBF1* alterations may be associated with improved survival outcomes in CRC, whilst the wild-type form could be an unfavorable prognostic marker (Figure 4).

**Figure 4:**
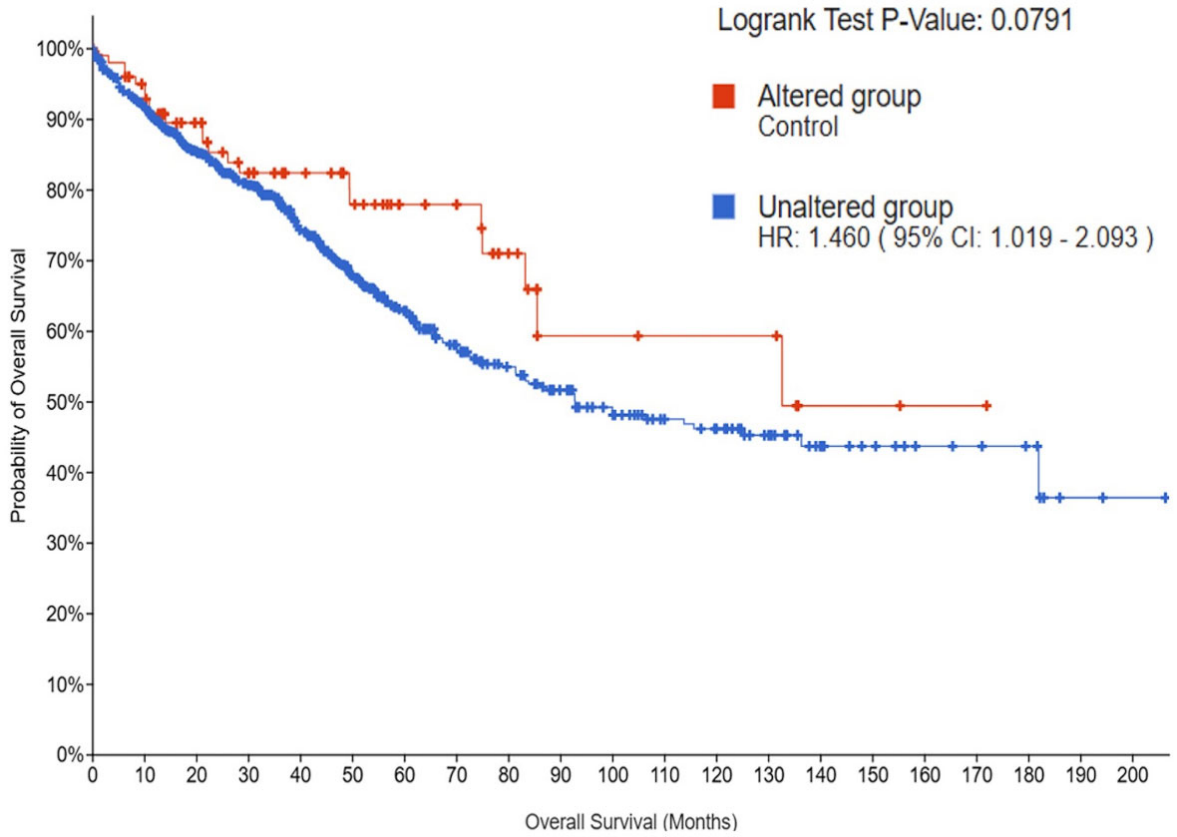
Kaplan-Meier plots of overall survival probability of colon cancer patients based on altered *SBF1* expression showed no significant association (P=0.0791), although there was an apparent trend toward better overall survival probability in patients expressing altered *SBF1* compared to those expressing wild-type *SBF1*. Plot created with the cBioPortal built-in statistical algorithms (26).

### Immunohistochemical analysis of SBF1 expression in CRC tissues

Immunohistochemical analysis was performed on formalin-fixed, paraffin-embedded (FFPE) colorectal tissue samples from 50 patients, obtained from King Fahad University Hospital. Due to duplication of slides from one patient, 49 tumor cases were included in the final analysis. Corresponding NATs were available for 30 cases, while 19 cases lacked NAT and were excluded from paired statistical comparisons. The cohort included both male and female patients across a wide age range, with tumors arising from various anatomical sites within the colon and rectum. The majority of cases were diagnosed as moderately differentiated adenocarcinoma, spanning multiple pathological stages (pT2-pT4, with variable nodal involvement).

Immunohistochemical staining revealed marked differences in SBF1 expression between malignant colorectal tissues and their corresponding normal adjacent tissues. Microscopic evaluation was performed at 200× and 400× magnifications, and staining intensity was manually scored on a semi-quantitative scale from 0 to 3 (0=no staining, 1=weak, 2=moderate, 3=strong).

Tumor tissues predominantly exhibited strong to moderate SBF1 staining, whereas normal adjacent tissues primarily showed weak or negative staining. Strong staining (+3) was observed in 25 of 49 tumor samples, while only 2 of 30 normal tissues demonstrated strong staining. Conversely, no tumor samples exhibited negative staining, whereas 10 normal tissues showed complete absence of SBF1 signal.

Comparative representative images of tumor and normal tissues are presented in Figure 5. In malignant tissues, SBF1 staining appeared more intense and widely distributed, localizing to multiple cellular compartments, including cytoplasmic, luminal, and basement membrane regions. In contrast, normal tissues displayed weak, focal, or absent staining with preserved glandular architecture.

**Figure 5:**
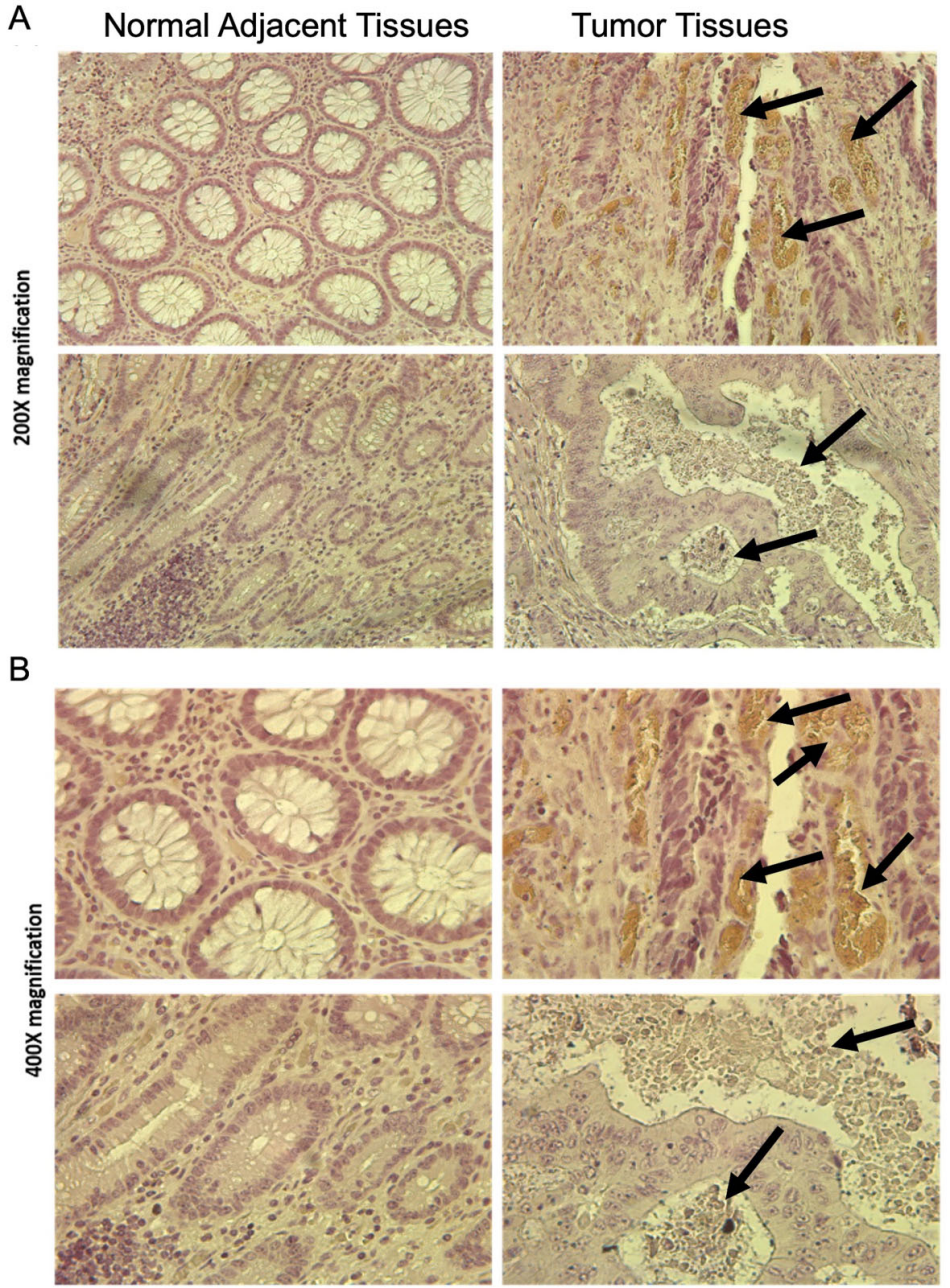
Photomicrographs of immunohistochemical staining of SBF1 in malignant and adjacent normal tissues from colon cancer patients captured at 200× (**A**) and 400× (**B**) magnifications. The left panels show normal colon tissue with well-organized glandular cells surrounded by supportive matrix. The right panels show malignant tissues with typical characteristics of malignant transformation, including a high nuclear/cytoplasmic ratio, nuclear molding, and dense chromatin. Malignant tissues show a much stronger SBF1 staining signal (brownish color; black arrows), which is distributed across multiple cellular locations, including basement membranes, cytosol, and luminal spaces.

Quantitative comparison of staining intensity scores demonstrated a significantly higher mean SBF1 expression in tumor tissues compared with NAT. When excluding cases without matched NAT, the mean staining score for tumor tissues was 2.30±0.13, whereas the mean score for normal tissues was 0.84±0.15.

The difference in SBF1 expression between tumor and normal tissues was highly significant (P<0.0001). The calculated *t*-value was 8.038 with 29 degrees of freedom, supporting a robust increase in SBF1 protein expression in colorectal tumor tissues relative to normal adjacent tissues (Figure 6).

**Figure 6:**
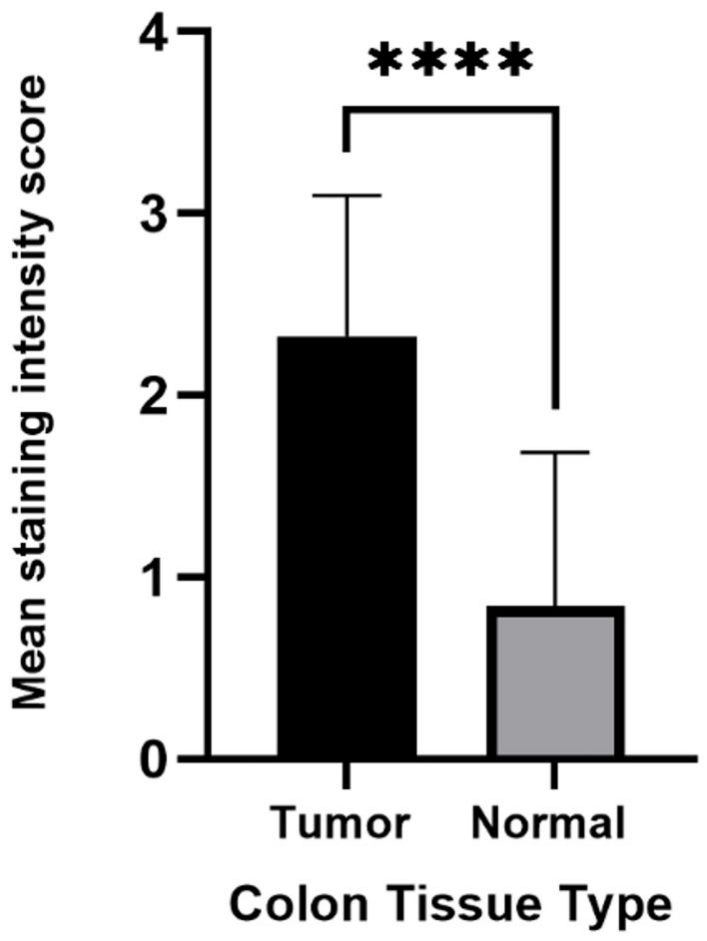
Comparison of the mean SBF1 staining intensity scores between tumor and normal adjacent tissues. Data are reported as means±SE. ****P<0.0001; Student's paired *t*-test.

To assess the association between CRC stage and staining intensity, stages 3 and 4 were classified as “severe” and stages 1 and 2 were classified as “less severe”. Scores 2 and 3 were classified as “high” and scores 0 and 1 as “low”. The 2×2 contingency table analysis indicated that all cases with advanced stages 3 or 4 had high intensity scores, while 6 of 18 patients with stages 1 or 2 had low intensity scores. Fisher's exact test revealed a significant association between cancer stage and SBF1 intensity scores (P=0.0191), indicating that patients with advanced CRC stages express higher levels of SBF1.

## Discussion

The present study provides new evidence supporting a potential role for Set Binding Factor 1 (*SBF1*; *MTMR5*) in CRC. To date, no experimental studies have directly investigated the expression or biological significance of SBF1 in colon cancer. The findings presented here address this gap by combining immunohistochemical evaluation of clinical specimens with complementary bioinformatics analyses, thereby offering an integrated view of SBF1 involvement in colorectal tumorigenesis and insights into its molecular functions.

Immunohistochemical analysis demonstrated that SBF1 protein expression was significantly elevated in malignant colorectal tissues compared with their normal adjacent counterparts. This consistent overexpression across tumor samples suggests that SBF1 may contribute to CRC development or progression. The observed increase in SBF1 staining intensity in tumor tissues, together with absent or weak expression in normal tissues, supports the notion that elevated SBF1 expression is tumor-associated rather than a feature of normal colonic epithelium.

These experimental observations were supported by *in silico* analyses using CRC datasets. Bioinformatics analysis revealed that patients harboring wild-type *SBF1* exhibited lower overall survival probabilities compared with those carrying altered forms of the gene, suggesting that *SBF1* status may be linked to disease outcome. Although this survival association did not reach statistical significance, hazard ratio analysis identified wild-type *SBF1* as a potential risk factor, indicating a possible biological relevance that warrants further investigation. Together, the tissue-based and computational findings converge to implicate *SBF1* in CRC biology.

While direct studies on *SBF1* in CRC are lacking, other members of the MTMR family have been examined in this malignancy. For example, *MTMR2* was reported to be significantly upregulated in multiple cancer types, including colon adenocarcinoma, based on large-scale transcriptomic analyses (28). Similarly, MTMR3 has been identified as a positive regulator of CRC cell proliferation and survival, where its silencing induced apoptosis and cell cycle arrest in CRC cell lines (29). Interestingly, bioinformatics analysis of *MTMR3* expression in CRC datasets revealed survival patterns similar to those observed for *SBF1*, with wild-type expression associated with poorer outcomes, further suggesting functional overlap or pathway convergence among MTMR family members.

In contrast, MTMR7 has been reported to function as a tumor suppressor in CRC. Loss of *MTMR7* expression was observed in a substantial proportion of colorectal tumors and was associated with enhanced Akt phosphorylation and increased cell proliferation (16). These contrasting roles among MTMR family members underscore the complexity of phosphoinositide signaling regulation in CRC. Despite sharing similar substrate specificities, MTMR proteins appear to exert divergent, context-dependent functions during tumorigenesis.

Autophagy is another biological process potentially linking MTMR proteins, including SBF1, to cancer. Several MTMR family members have been shown to suppress autophagy, while downregulation of *MTMR3* and *MTMR5* has been associated with autophagy induction (30-32). In CRC, induction of autophagy following *MTMR3* silencing was associated with reduced cell proliferation (33). Although autophagy was not directly assessed in the present study, these findings suggest a potential mechanistic framework through which *SBF1* overexpression may influence CRC cell behavior.

Although SBF1 interaction partners were identified through protein-protein interaction analysis, correlation between SBF1 expression and the levels of these interacting proteins in CRC samples was not evaluated in this study. Future investigations integrating transcriptomic and/or proteomic analysis may help clarify these relationships. However, high confidence scores were provided by the “string” portal for SBF1 interactions with multiple partners in the network.

Collectively, the data presented here provide the first experimental evidence that *SBF1* is significantly overexpressed in CRC tissues and that its level was associated with tumor stage. This supports SBF1 potential involvement in tumor development and patient outcomes. However, functional studies using CRC cell lines and *in vivo* models are required to elucidate the precise molecular mechanisms by which SBF1 contributes to colorectal tumorigenesis.

## Data Availability

All datasets generated and analyzed in the present study have been deposited in the Figshare repository. Links to bioinformatics datasets retrieved from cbioportal can be accessed through https://doi.org/10.6084/m9.figshare.31491037. De-identified raw staining scores and the processed datasets supporting the findings of this study are available at https://doi.org/10.6084/m9.figshare.31500085.

## Supplementary Materials

Click to view [pdf].
