# Supplementary material for: Set binding factor 1 (SBF1/MTMR5) as a potential novel target in colorectal cancer: an exploratory immunohistochemical and bioinformatics study
Source: Braz J Med Biol Res. 2026 Oct 12;59:e15598. doi: 10.1590/1414-431X2026e15598 (PMC13660866; doi:10.1590/1414-431X2026e15598)
Supplement: Supplementary file 1 [file 1414-431X-bjmbr-59-e15598-suppl.pdf]

Figure S1. Genetic analysis of *SBF1* in colorectal cancer using the cBioPortal database. A, Genetic alteration frequency of *SBF1*, indicating that it is genetically altered in 5% of samples. B, Alteration frequencies of *SBF1* across multiple colorectal cancer datasets. C, Mutation sites of *SBF1*. The figure was provided by cBiportal: cBioPortal for Cancer Genomics. SBF1 genetic alterations, expression and survival analysis in colorectal cancer (TCGA dataset). 2025 [cited 22 Jun 2025]; Available from: <https://www.cbioportal.org/results/oncoprint?cancer_study_list=coadread_dfci_2016%2Ccoadread_genentech%2Ccoadread_tcga%2Ccoadread_tcga_pub%2Ccoadread_tcga_pan_can_atlas_2018%2Ccoadread_mskcc%2Ccoadread_cass_2020%2Ccoadread_mskresistance_2022%2Ccrc_apc_impact_2020%2Ccrc_dd_2022%2Ccrc_sysucc_2022%2Ccrc_msk_2017%2Ccoad_cptac_gdc%2Ccoad_caseccc_2015%2Ccoad_tcga_gdc%2Ccoad_cptac_2019%2Ccoad_silu_2022&Z_SCORE_THRESHOLD=2.0&RPPA_SCORE_THRESHOLD=2.0&profileFilter=mutations%2Cstructural_variants%2Cgistic%2Ccna&case_set_id=all&gene_list=SBF1&geneset_list=%20&tab_index=tab_visualize&Action=Submit>.

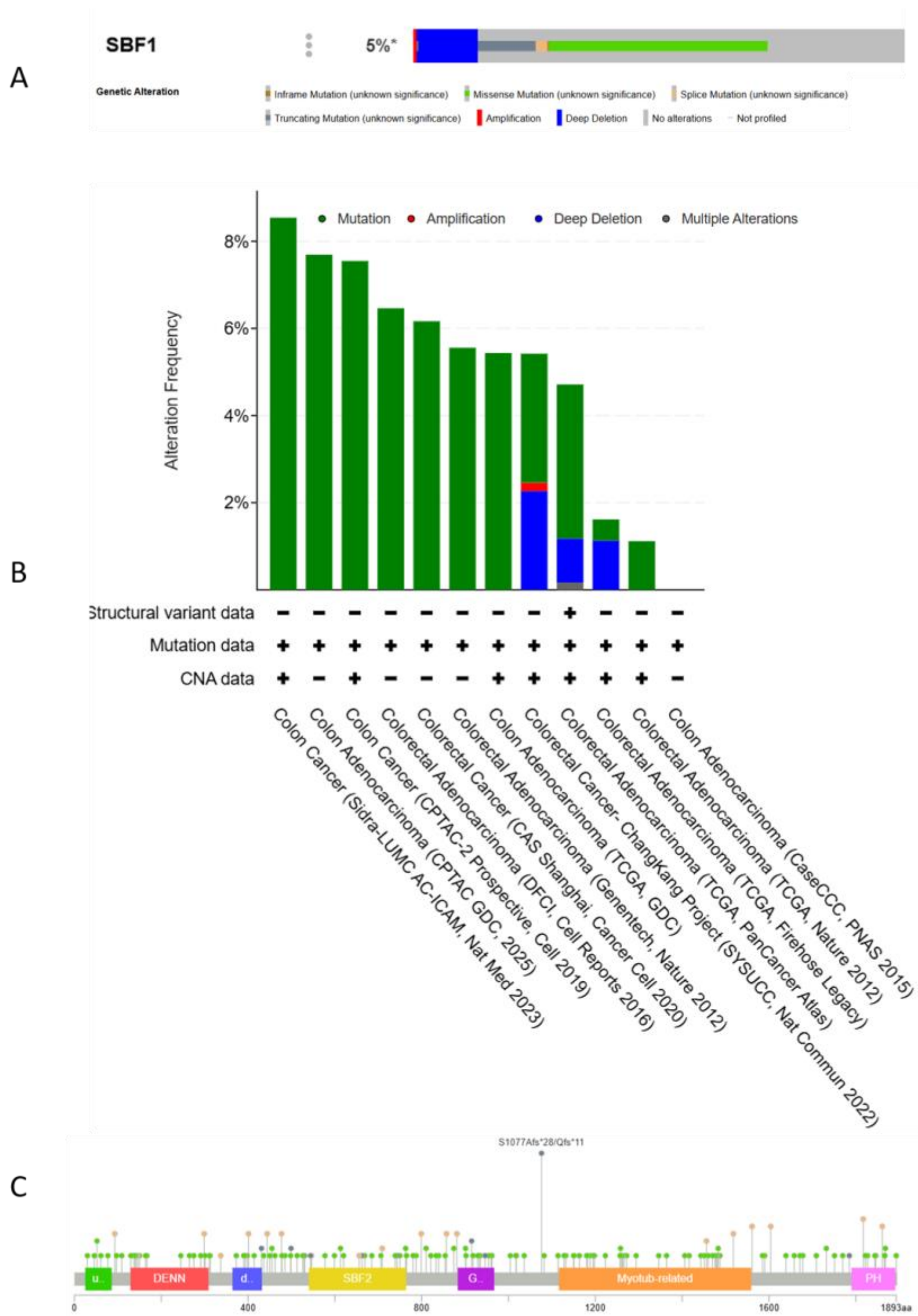

Table S1. Gene Ontology enrichment terms, including biological processes and molecular functions of SBF1 network.

Protein Network	Biological processes	Molecular functions
SBF1, MTMR2, MTMR1, FZR1, KMT2A, RAB21, MTMR6, FIG4, SH3TC2, SUV39H1, and FGD4	Phosphatidylinositol dephosphorylation	Phosphatidylinositol-3,5-bisphosphate phosphatase activity
	Dephosphorylation	Phosphatidylinositol-3-phosphatase activity
	Phosphatidylinositol biosynthetic process	Phosphatidylinositol-3,5-bisphosphate 3-phosphatase activity
	Regulation of phosphatidylinositol dephosphorylation	Phosphatase activity
	Positive regulation of early endosome to late endosome transport	Phosphoprotein phosphatase activity
	Negative regulation of myelination	
	Myelination	

Table S2. Gene ontology terms, the supporting evidence, and the confidence score of protein partners of SBF1.

Protein partners	Gene Ontology annotation	Type of evidence	Score
MTMR2	**Molecular functions include:**<br>Phosphatidylinositol-3-phosphate phosphatase activity<br>Protein binding<br>Hydrolase activity<br>Phosphatidylinositol-3,5-bisphosphate 3-phosphatase activity<br>**Biological processes include:**<br>Myelination<br>Phosphatidylinositol dephosphorylation<br>Negative regulation of receptor internalization<br>Negative regulation of endocytosis<br>Negative regulation of excitatory postsynaptic potential<br>Dendritic spine maintenance<br>Positive regulation of early endosome to late endosome transport<br>Negative regulation of receptor catabolic process	Homology<br>Text-mining<br>Databases<br>Experiments	*0.993
MTMR1	**Molecular functions include:**<br>Phosphatidylinositol-3-phosphate phosphatase activity<br>Hydrolase activity<br>Protein homodimerization activity<br>Phosphatidylinositol-3,5-bisphosphate 3-phosphatase activity<br>Protein binding<br>**Biological Processes include:**<br>Lipid metabolic process<br>Phosphatidylinositol biosynthetic process<br>Phosphatidylinositol dephosphorylation<br>Macroautophagy	Homology<br>Text-mining<br>Experiments<br>Co-expression	*0.860
FZR1	**Molecular functions include:**<br>Protein binding<br>Anaphase-promoting complex binding<br>Ubiquitin ligase activator activity<br>**Biological Processes include:**<br>Positive regulation of anaphase-promoting complex-dependent catabolic process<br>Regulation of meiotic nuclear division<br>Positive regulation of ubiquitin protein ligase activity<br>DNA synthesis involved in mitotic DNA replication<br>DNA damage response<br>Protein ubiquitination<br>Cell division<br>Negative regulation of cellular senescence	Text-mining<br>Co-expression	*0.841
KMT2A	**Molecular functions include:**<br>DNA binding<br>Chromatin binding<br>Histone H3K4 methyltransferase activity<br>**Biological processes include:**<br>Epigenetic regulation of gene expression<br>Negative regulation of fibroblast proliferation<br>Circadian regulation of gene expression<br>Embryonic hemopoiesis<br>Transcription initiation-coupled chromatin remodeling<br>Positive regulation of DNA-templated transcription<br>Positive regulation of transcription by RNA polymerase II<br>Chromatin remodeling	Text-mining<br>Experiments<br>Co-expression	*0.755
RAB21	**Molecular Functions Include:**<br>GTPase activity<br>GTP binding<br>GDP binding<br>Protein binding<br>**Biological processes include:**<br>Positive regulation of early endosome to late endosome transport<br>Intracellular protein transport<br>Protein transport<br>Regulation of exocytosis<br>Positive regulation of receptor-mediated endocytosis	Text-mining<br>Databases<br>Experiments<br>Co-expression	*0.720
MTMR6	**Molecular functions include:**<br>Phosphatidylinositol-3-phosphate phosphatase activity<br>Phosphatidylinositol-3,5-bisphosphate 3-phosphatase activity<br>Protein serine/threonine phosphatase activity<br>Protein binding<br>**Biological processes include:**<br>Phosphatidylinositol dephosphorylation<br>Endocytosis<br>Protein dephosphorylation	Homology<br>Text-mining<br>Co-expression	0.614

*Strong interaction probability with score ≥0.7.

Table S3. Gene ontology terms, the supporting evidence, and the confidence score of protein partners of SBF1.

Protein partners	Gene Ontology annotation	Type of evidence	Score
FIG4	**Molecular functions include:**<br>Phosphatidylinositol-3-phosphate phosphatase activity<br>Phosphatidylinositol-3,5-bisphosphate 5-phosphatase activity<br>Phosphatidylinositol-4,5-bisphosphate 5-phosphatase activity<br>Phosphatidylinositol-3,4,5-trisphosphate 5-phosphatase activity<br>Phosphatidylinositol-4-phosphate phosphatase activity<br>**Biological processes include:**<br>Early endosome membrane<br>Late endosome membrane<br>Recycling endosome<br>Intracellular membrane-bounded organelle	Text-mining	0.611
SH3TC2	**Molecular functions include:**<br>Protein binding<br>**Biological processes include:**<br>Myelination in the peripheral nervous system<br>Regulation of intracellular protein transport<br>Regulation of ERBB signaling pathway<br>Regulation of endocytic recycling	Text-mining	0.610
SUV39H1	**Molecular functions include:**<br>RNA polymerase II transcription regulatory region sequence-specific DNA binding<br>Chromatin binding<br>Histone deacetylase activity<br>Methyltransferase activity<br>Histone methyltransferase activity<br>Histone H3K9 methyltransferase activity<br>**Biological processes include:**<br>Negative regulation of cell cycle<br>Negative regulation of DNA-templated transcription<br>DNA damage response<br>Regulation of multicellular organism growth<br>Regulation of cellular senescence<br>Chromatin organization<br>Cell differentiation<br>Negative regulation of gene expression, epigenetic<br>Negative regulation of DNA-templated transcription	Text-mining<br>Experiments<br>Co-expression	0.597
FGD4	**Molecular functions include:**<br>Actin binding<br>Guanyl-nucleotide exchange factor activity<br>Small GTPase binding<br>**Biological Processes include:**<br>Cytoskeleton organization<br>Regulation of cell shape<br>Regulation of small GTPase mediated signal transduction	Text-mining	0.585

Strong interaction probability with score ≥0.7.
